# Microbiota and Microglia Interactions in ASD

**DOI:** 10.3389/fimmu.2021.676255

**Published:** 2021-05-25

**Authors:** Marcela Davoli-Ferreira, Carolyn A. Thomson, Kathy D. McCoy

**Affiliations:** Department of Physiology and Pharmacology, Snyder Institute of Chronic Diseases, Cumming School of Medicine, University of Calgary, Calgary, AB, Canada

**Keywords:** neurodevelopmental disorders, inflammation, dysbiosis, microbial metabolites, autism spectrum disorder (ASD), microglia, microbiome

## Abstract

Autism spectrum disorders (ASD) are serious, highly variable neurodevelopmental disorders, commonly characterized by the manifestation of specific behavioral abnormalities, such as stereotypic behaviors and deficits in social skills, including communication. Although the neurobiological basis for ASD has attracted attention in recent decades, the role of microglial cells, which are the main resident myeloid cell population in the brain, is still controversial and underexplored. Microglia play several fundamental roles in orchestrating brain development and homeostasis. As such, alterations in the intrinsic functions of these cells could be one of the driving forces responsible for the development of various neurodevelopmental disorders, including ASD. Microglia are highly sensitive to environmental cues. Amongst the environmental factors known to influence their intrinsic functions, the gut microbiota has emerged as a central player, controlling both microglial maturation and activation. Strikingly, there is now compelling data suggesting that the intestinal microbiota can play a causative role in driving the behavioural changes associated with ASD. Not only is intestinal dysbiosis commonly reported in ASD patients, but therapies targeting the microbiome can markedly alleviate behavioral symptoms. Here we explore the emerging mechanisms by which altered microglial functions could contribute to several major etiological factors of ASD. We then demonstrate how pre- and postnatal environmental stimuli can modulate microglial cell phenotype and function, underpinning the notion that reciprocal interactions between microglia and intestinal microbes could play a crucial role in ASD aetiology.

## Background

Autism spectrum disorders (ASD) include a range of neurodevelopmental disorders, commonly characterized by repetitive behaviours, as well as impaired social skills, including verbal and nonverbal communication (1). These behavioral symptoms develop in early childhood and persist throughout life. In recent decades, there has been a major surge in ASD incidence globally (2). Although the precise aetiologies of ASD are complex, and remain to be fully understood, recent evidence points to abnormal synaptic development and function, and/or aberrant immune responses, as potential drivers of ASD symptoms (3–6). Notably, microglial cells participate in these physiological processes and have been strongly associated with ASD development (7–10).

Microglia are the main resident immune cells of the central nervous system (CNS), providing the tissue with innate immune sensing, inflammatory effector functions and tissue repair. As such, they are the main producers of proinflammatory mediators in the context of neuroinflammation (11). Although immunomodulatory roles for microglia in neuroinflammatory and neurodegenerative diseases have been widely described, immune modulation is only one of an extensive array of discrete microglial functions. During CNS development, microglia regulate the number and strategic positioning of neurons and shape neuronal connectivity (10, 12). Moreover, they support gliogenesis and myelination (10, 12–15). Given both their immune and developmental functions, it would be attractive to propose that microglial dysfunction could contribute to neurodevelopmental disorders; either by influencing disease development or driving behavioral symptoms. However, the specific roles that microglial cells play in ASD pathophysiology are still controversial. Although several studies show that autistic individuals suffer from ongoing neuroinflammatory processes, characterized by microglial activation in several discrete regions of the brain (16–19), others dispute the significance of this and suggest that microglia may be intrinsically dysfunctional in their resting state, following a prenatal disruption to homeostatic brain development (20, 21). In this review, we explore both well-established and emerging literature and discuss perspectives on the role’s microglia may play in the development of ASD; both in the context of abnormal immune signaling and altered neuronal connectivity. Given the vast array of peripheral factors that can modulate microglial maturation and function, we further discuss how perturbations in these extrinsic signals, particularly the gut microbiota, might promote microglial dysfunction in the context of the neurodevelopmental disorders.

## Microglia: Origin and Physiological Functions in the Brain

**Figure 1:**
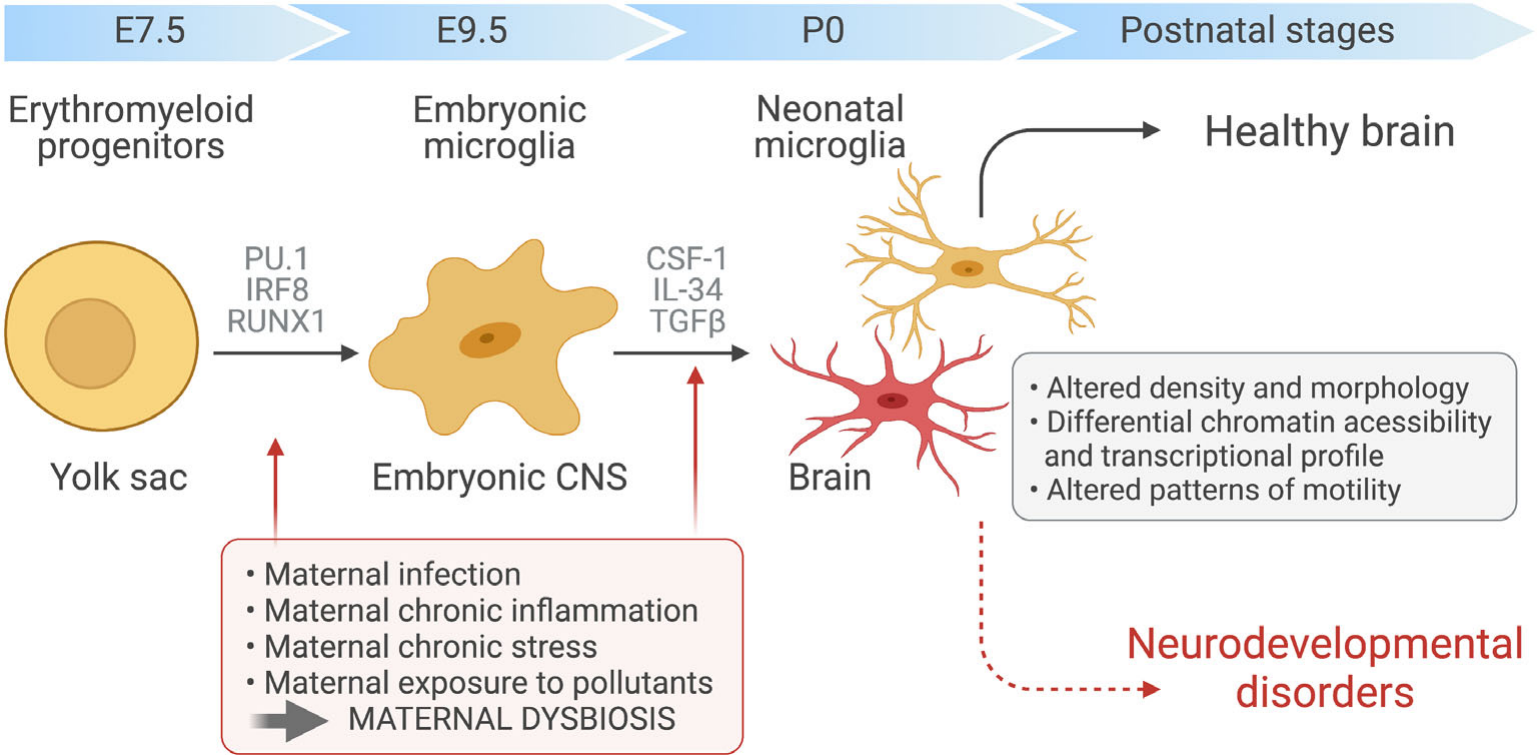
Maternal immune activation and dysbiosis in microglial development. Yolk sac-derived erythroid progenitors differentiate into microglia progenitors, *via* Runx1, PU.1-and IRF8-dependent pathways, that then migrate and colonize the developing brain at around embryonic day 9.5. After microglial seeding of the embryonic CNS parenchyma and subsequent proliferation during prenatal and postnatal stages, factors such as CSF-1, IL-34 and TGF-β promote microglia terminal differentiation. Maternal chronic inflammatory diseases, maternal infection and exposure to environmental factors, such as pesticides and pollution, can induce immune activation during pregnancy and dramatic changes in maternal microbiota. These alterations can disrupt the normal prenatal microglia development, maturation and induce microglial epigenetics alterations, affecting the developing fetal brain and leading to ASD development.

Microglia are a highly specialised population of myeloid cells that inhabit the healthy CNS parenchyma, representing 5–12% of all cells in the CNS (22). Unlike the other cell types that coinhabit the CNS, microglia are not derived from the neuroectodermal germ layer. Rather, microglial ontogeny has been traced to erythromyeloid precursors, which differentiate into microglial progenitors in the yolk sac during embryogenesis (23, 24). At this stage, differentiation is critically controlled by the transcription factors Pu.1 and Irf8 with other transcription factors, such Runx1 and Jun, also providing a supporting role (21, 23, 24). On day 9.5 after conception (E9.5), microglial progenitors leave the yolk sac to seed the developing CNS in one single wave (22–24). Following an initial burst of proliferation and differentiation, mature microglia then colonize the parenchyma where they persist throughout the life of the host (**Figure 1**). There, within the healthy CNS, microglial numbers are maintained by gradual self-renewal, independently from the recruitment of any other hematopoietic myeloid cells or progenitors (11, 24, 25).

Within the CNS parenchyma, microglia are imprinted by local environmental cues. Microglial differentiation and maintenance are strongly dependent on their expression of colony-stimulating factor 1 receptor (CSF1R), as well as the two main CSF1R ligands, CSF1 and IL-34 (**Figure 1**). Depleting either of these ligands reduces microglial cell abundance throughout the CNS. Moreover, the CNS of adult CSF1R-deficient mice are virtually devoid of all microglia (26, 27). By driving a microglia-specific gene signature, TGFβ signaling has recently also been shown to be indispensable for microglia maturation. The marker genes induced by TGFβ, which include *Tmem119, Sall1, Tgfbr1*, and *P2ry12*, can readily distinguish microglia from bone marrow-derived macrophages (28).

As tissue-resident macrophages, microglial cells are responsible for the continuous immunosurveillance of the CNS. Inflammatory insults induced by invading pathogens or local injuries trigger their production of immune mediators (29–31). These pathways also facilitate increased phagocytosis of cellular debris and/or pathogens (32). Previously, microglia were thought to be inactive during homeostasis and only activated in response to pathological insults. However, in addition to their “canonical” innate immune functions, recent findings suggest that microglia are intimately involved in CNS development through organising neuronal patterning and fine-tuning synaptic connections (13, 22).

During embryogenesis, microglia are the first glial cells to populate the developing CNS. In this early neurodevelopmental phase, they control neurogenesis by releasing neurotoxic or neurotrophic factors that orchestrate the survival, differentiation or apoptosis of neuronal progenitors (33–35). The survival-enhancing role of microglia is supported by findings showing that proliferation and survival of these progenitors is higher when they are co-cultured with microglia than when cultured alone (34). On the other hand, microglial respiratory bursts generate superoxide ions, which trigger the apoptosis of Purkinje cells in the postnatal cerebellum (33). Thus, through the selective release of neurotoxic or neurotrophic factors, microglia can shape the neuronal landscape.

In addition to modulating neurogenesis, microglia play important roles in the development and differentiation of neuronal circuits. From an early stage in postnatal neurodevelopment, microglia eliminate redundant neurons that do not establish functional circuits. Moreover, microglia modulate immature neuronal circuits by engulfing and eliminating dendritic spines at the synapse (10). This process, known as synaptic pruning, is critically important for the normal formation of synapses. Its disruption results in several neuronal abnormalities; including impaired functional connectivity, modifications to dopaminergic circuits, and an imbalance of the excitation-to-inhibition ratio in the cortex (9, 10, 36). Importantly, abnormal synaptic pruning in the CNS of the neonate, or even the developing fetus, could be important in the aetiology of ASD, as discussed later.

Finally, there is now cumulating evidence that microglial cells modulate synaptic plasticity, and subsequently, learning and memory (37–39). This is not only important during early developmental stages as depleting microglia from the CNS of adult mice also results in impaired synaptic plasticity and deficits in learning and memory (13, 40–42). Similar phenotypes are observed when microglia are unable to produce brain-derived neurotrophic factor (BDNF), as shown using conditional and inducible BDNF depletion under the CX_3_CR1 promotor (42)

Thus, although microglia have several well-defined roles in neuroinflammation, it is becoming increasingly evident that they also shape neuronal survival and connectivity during development, interpret changes in the local milieu and modulate circuit formation accordingly (11, 43).

## Microglia in ASD

To date, microglial cell participation in ASD and other neurodevelopmental disorders has been only speculated. While the causes of ASD are incompletely understood, some of the main symptoms, such as impairment in multisensory processing and integration, have been linked to defects in neurogenesis and the strategic positioning of neurons during CNS development, abnormal synaptic pruning and an altered neuronal excitation/inhibition ratio (44). Additionally, systemic and central inflammation may also be intrinsically involved in the pathogenesis of ASD and several other neurological disorders (45, 46). Considering both the physiological roles microglia play in regulating neurogenesis, neuronal migration and synaptic pruning, and their immunomodulatory roles in the CNS, it seems entirely plausible that aberrant microglial function may be a driving force in the pathogenesis of ASD.

Vargas et al. were the first to show an inflammatory phenotype in post-mortem brains from ASD individuals. In this pioneer work, neuropathologic analysis showed increased microglial activation, characterized by elevated expression of MHC class II, throughout the cerebral and cerebellar cortices in individuals with ASD. Moreover, increased expression of pro- and anti-inflammatory factors, such as such as CCL2, IL-6 and TGF-β, were observed in both the brain and cerebrospinal fluid (CSF) (16). Similar studies have reported increased expression of TNF-α, IL-6, IL-8, GM-CSF, and more recently, IL-18 and IL-37, in post-mortem brain tissue and CSF of children with ASD, suggesting a heightened immune response with associated localized brain inflammation (47–49). Consistent with this apparent microglial and astrocyte immune dysregulation, genome-wide analysis of brain tissue from ASD individuals showed enrichment of markers related to activated microglia and expression of genes associated with “immune and inflammatory” gene ontology categories, compared to neurotypical controls (50, 51). These changes in microglial activation markers in ASD brains were also accompanied by changes in microglial morphology, density and spatial localization (18, 52, 53). Not only do microglia have an increased density throughout the cerebral and cerebellar cortices of ASD patients, but they exhibit cell body enlargement, as well as process retraction and thickening. Filopodia also extend from the processes of ASD-associated microglia (18, 54). The putative microglial dysfunction detected in post-mortem samples has now been further confirmed using *In Vivo* Positron Emission Tomography (PET). In this study, which focused on young adults with ASD, a radiotracer specific for activated microglia and astrocytes was used to show a marked activation of these cells in several discrete regions of the brain (17).

The combination of neuropathological analyses of post-mortem human brain samples and PET scanning of live human ASD patients has provided compelling evidence to suggest that aberrant microglia and astrocyte immune activation is a common hallmark of ASD. However, due to the small number of samples evaluated, variations in genetic backgrounds, lifestyle choices, medication use and socioeconomic status, more studies are required. In the case of post-mortem studies, the cause of death could also impact brain inflammation. Moreover, it remains to be established whether microglial activation is a secondary effect of aberrant brain development or whether microglia play a causative role in the initiation or manifestation of ASD. For that reason, environmental and genetic rodent models are widely employed to explore the range of contributions microglia make to ASD pathogenesis, including their effects on neuronal migration, neurotransmission, brain anatomy and inflammation.

In rodents, genetic manipulation of microglia can profoundly alter CNS function, culminating in behavioral abnormalities resembling those found in ASD. For example, mice lacking the gene encoding CX_3_CR1 exhibit ASD-like behaviours, including social deficits (9, 10). Also known as the fractalkine receptor, CX_3_CR1 is a chemokine receptor that facilitates direct contact between microglial cells and CX_3_CL1 (fractalkine)-expressing neurons; an interaction known to suppress microglial cell activation and IL-1β production following peripheral immune stimulation (55). Signaling through the fractalkine/CX_3_CR1 axis is required for the optimal recruitment of microglia to specific CNS locations during embryogenesis (56). As such, *Cx3cr1*-deficient mice have fewer microglia present in the CNS during early postnatal development, resulting in altered synaptic pruning and subsequent deficits in neuronal connectivity throughout life (9, 10). Similar to CX_3_CR1, microglial expression of immunoglobulin superfamily-member, triggering receptor expressed on myeloid cells 2 (TREM2), is fundamental for synaptic pruning during prenatal neurodevelopment (57). TREM2 signalling transduction has a central role in promoting microglial activation (11) and variants in *TREM2* have been linked to different types of neurological diseases, including multiple sclerosis, Parkinson’s and Alzheimer’s diseases (58–62). Recent studies in mice show that the absence of this receptor results in defective remodelling of neuronal synapses, dysregulated excitatory/inhibitory neurotransmission, impaired neuronal connectivity and behavioral defects reminiscent of ASD (57). The expression of TREM2 was also significantly reduced in post-mortem brain tissue from individuals with ASD compared to neurotypical controls. This ASD-associated reduction in TREM2 expression was most prominent in samples collected from patients with severe symptoms, showing a negative correlation between TREM2 levels and ASD severity (57).

Recent studies have also shown that elevating protein synthesis, induced exclusively in microglia *via* overexpression of the translation initiation factor eIF4E, is sufficient to impair synaptic formation and drive the manifestation of ASD-like behaviors in young mice (63). Indeed, mutations that inactivate negative regulators of translation, such as in PTEN (phosphatase and tensin homolog), TSC1/2 (tuberous sclerosis complex 1/2), and FMR1 (fragile X mental retardation protein), are thought to cause ASD in a proportion of patients (64–68). Xu and collaborators suggested that defects in these ubiquitously expressed genes can alter microglial cell function sufficiently to drive ASD. In addition to an increased phagocytic potential, these microglia exhibit reduced mobility and impaired synaptic pruning, culminating in higher synapse density and higher excitatory neurotransmission compared to wild type mice, ultimately driving the development of ASD-like behaviors (63). Similar to the phenotype observed in mice that overexpressed eIF4E, the frequency, phenotype and function of microglia in the prefrontal cortex, hippocampus and striatum of *Pten*-deficient mice was substantially altered when compared with their wildtype littermates (63, 69, 70). Collectively, these studies add further weight to the hypothesis that aberrant microglial cell functions may help to drive the pathophysiology and behavioural symptoms associated with ASD.

Other models of autism in which risk genes are depleted in rodents to model symptomatic ASD variants, such as Rett syndrome and fragile X syndrome, are also related to microglial-dependent synaptic modulation. In *Fmr1*-deficient mice, a model of fragile X syndrome, microglia are increased in terms of size and abundance compared to those from wild-type littermates, and these physiological changes were associated with reduced microglial-mediated synaptic pruning (63, 71). As it is caused by loss-of-function mutations in the gene encoding methyl-CpG binding protein 2 (MECP2), Rett syndrome is modeled using variations of *Mecp2*-deficient mice. The specific deletion of *Mecp2* in murine microglial cells triggers an overproduction of glutamate, altering neuronal morphology and impeding the formation of synapses (72). Abnormal microglia–synapse interactions, and increased expression of inflammatory genes in macrophages and microglia, were also observed in mice lacking *Mecp2* (13, 73). These studies imply a pathological role for microglial cell dysfunction in ASD, apparently without the context of neuroinflammation. However, since both resting and activated microglia are able to secrete cytokines, neurotoxic and neurotrophic factors, as well as other soluble factors that have been implicated in ASD, it is possible that microglia use these mediators to influence a diverse range of neuronal functions and sculpt synaptic connections (19, 20, 74).

In addition to genetic factors, environmental factors also modify microglia function, affecting brain development, synaptic connectivity and CNS immune responses (75). Indeed, the behavioural abnormalities that are observed in mouse models in which environmental risk factors are the driving forces behind ASD development, such as the maternal immune activation (MIA) model, are similar to those induced by genetic modification (75, 76). The MIA model, in which pregnant mice are challenged with polyinosinic-polycytidylic acid [Poly(I:C)] or lipopolysaccharide (LPS) during embryonic development (E9–12), was developed based on numerous epidemiological studies that have linked prenatal infection in humans to the development of several neurological disorders, including ASD, in the offspring (13, 77).

MIA remotely triggers the induction of multiple cytokines in the fetal brain of rodents, subsequently leading to abnormal neurodevelopment. Due to their rapid ability to respond to inflammatory signals and their role in modulating neuronal function and connectivity, microglial cells have been implicated in driving this disorder (78). However, contradicting results cloud the ability to draw clear conclusions on how and when MIA shapes microglial functions in the developing offspring. While some studies showed increased expression of microglial activation markers in adult offspring exposed to MIA *in utero*, others did not uncover any postnatal differences in microglial phenotype as a consequence of MIA. The most consistent microglial changes were found during pre- or perinatal developmental stages, suggesting that transient perturbations in microglial function might have life-long effects on neuronal patterning, functional connectivity and behaviour (79–82). Supporting this hypothesis, studies using genome-wide chromatin accessibility assays revealed a series of temporally distinct developmental stages, both pre- and perinatal, during which the susceptibility of microglia to immune mediators and other environmental cues was increased (21, 24). Microglia from newborns exposed to maternal immune activation showed an untimely downregulation of genes that are typically expressed during this early stage of development, such as *Spi1* (the gene encoding Pu.1) and *Irf8*, and instead exhibited a transcriptional phenotype more akin to that of adult microglia (21, 24). Although these alterations were transient, the authors suggested that accelerated microglial maturation could have sufficient detrimental consequences in the developing brain to induce and maintain neurological disorders that continue long after the microglia phenotype is restored (21).

Together, these data strengthen the notion that microglia can play a fundamental role in driving neurodevelopmental disorders, including ASD, *via* their effects on neuroimmune pathways, synaptic remodelling, neuronal survival and connectivity. Understanding the main factors that induce microglial dysfunction and identifying developmental timepoints when the CNS is most susceptible to the impacts of microglial dysregulation would help to identify novel therapeutic targets and prophylactic strategies to better treat or prevent ASD.

## Environmental Factors Influencing ASD: Focus on Microbiota-Microglia Modulation

Although genetic factors can majorly influence the risk of ASD development, epidemiological and preclinical studies estimate that 50% of ASD pathophysiology is driven by non-heritable factors, suggesting that environmental factors may play an equally prominent role (83, 84). However, while progress has been made towards gaining an understanding of the genetic components that drive ASD, environmental risk factors are less understood. Several recent studies suggest that prenatal, perinatal and postnatal factors act synergistically to induce the development of ASD (85). Maternal diet and lifestyle, as well as exposure to infection, environmental chemicals and drugs during critical periods of CNS development, can induce various congenital malformations, culminating in a latent and long-term impact on brain function, and enhancing the risk of ASD development in the offspring (86–90). Many environmental components that are vertically transmitted *via* mother-to-child interactions can influence brain development during peri- and postnatal periods, whilst horizontally transferred external factors, i.e. those that are not dependent on the maternal interface, have the capacity to interfere with the maintenance and progression of ASD symptoms (91, 92).

### The Role of the Gut Microbiota in ASD Development and Maintenance

Intestinal microbes are intimately involved in integrating the various environmental factors, such as diet, environment, sex, age and genetic background, which subsequently impact host immune responses (93). Remarkably, gastrointestinal (GI) dysfunction is one of the most prominent comorbidities in ASD patients, with 23-70% of the individuals developing symptoms associated with the GI tract, including abdominal discomfort, irritated bowel syndrome, chronic diarrhea and/or constipation (32, 94). Moreover, variations in the composition and richness (diversity) of the gut microbiota have been observed in children with ASD compared to neurotypical controls, with several reports of increased proportions of *Clostridium, Suterella, Ruminococcus* and *Lactobacillus* and lower abundances of *Bifidobacterium, Akkermansia, Blautia* and *Prevotella* (95–100).

Based on the apparent dysbiosis observed in ASD individuals, numerous cross-sectional studies have investigated whether exposure to antibiotics during different developmental stages could play a causative role in triggering the onset of ASD (101–106). Although current data are conflicting and inconclusive, the most consistent data obtained from the larger cohort studies indicate that the use of specific classes of antibiotics during early life may marginally increase the risk of ASD development (103, 104, 107).

The studies described above have been useful in identifying associations between ASD and the GI tract, particularly with dysbiosis of the gut microbiota. In an open-label trial, fecal microbiota transplant (FMT) therapy from neurotypical control donors to ASD patients significantly increased bacterial diversity and improved irritability, communication skills and sociability (108, 109). Thus, the gut microbiota may contribute to the behavioural symptoms associated with ASD.

In mice, the induction of dysbiosis – for example using dietary modulation, antibiotics or gnotobiotic models – can aggravate both genetic and environmental models of ASD (110). Germ-free (GF) mice have significant social impairments compared to specific pathogen-free (SPF) mice, as do mice that receive antibiotics postnatally (111–113). Moreover, transferring dysbiotic gut microbiota from ASD donors was sufficient to induce further social deficits and increase repetitive behaviours in GF mice, compared to mice that received an FMT from neurotypical control donors (114). These data demonstrate that ASD-like behaviours can be transferred by the microbiota of ASD patients.

Dysbiosis of the commensal microbiota has also been observed in the offspring of mice exposed to the MIA model, apparently contributing to barrier permeability and behavioural changes. In particular, MIA-exposed offspring had reduced levels of *Bacteroides fragilis* in the gut compared to controls. Importantly, reintroducing *Bacteroides fragilis* to the GI tract of these mice was sufficient to restore GI function and improve the neurological symptoms related to ASD (115, 116). Together, these data demonstrate that the commensal microbiota may be crucial for the programming and presentation of neurotypical behaviours.

It is important to consider that the studies outlined above predominantly focus on how the microbiota of the individual impacts the progression of ASD. While ASD symptoms were alleviated following FMT from neurotypical donors, the effects were transient and do not constitute a cure. As ASD is established early in development, including during embryogenesis, it seems likely that environmental factors experienced by a mother during gestation may play an equally, if not more important role. It is therefore not surprising that in rodent models of ASD, the maternal gut microbiota has also been implicating in remotely conditioning neurodevelopment, subsequently leading to ASD-like behavioural changes in the offspring. In mice, the presence of bacteria that can drive Th17 cell induction, such as segmented filamentous bacteria (SFB), is required to induce ASD development in the offspring of dams exposed to MIA (117, 118). Moreover, while the maternal microbiota is essential for normal fetal neurodevelopment (119), dysbiosis induced in response to altered diet and stress during pregnancy has also been increasingly linked to aberrant brain development and behavioural abnormalities in murine offspring (110, 119, 120). Thus, the vertical transfer of microbial molecules or microbially-induced intermediates, may alter brain function in the developing offspring, ultimately triggering the development of ASD-like behaviours.

A conclusive link between the maternal microbiota and ASD development in human patients has yet to be established. However, mothers of children with ASD often present with compositional differences in their gut microbiota, including increased levels of *Proteobacteria*, *Alphaproteobacteria*, *Moraxellaceae*, and *Acinetobacter*, when compared to mothers of healthy, neurotypical children (121). Moreover, meta-analyses of large cohort studies suggest prenatal exposure to different classes of antibiotics could contribute to the development of ASD (122, 123). However, data linking prenatal antibiotic exposure to ASD development in the offspring are highly controversial, and these studies neglect to evaluate the impact that antibiotic treatments have on the composition of the maternal microbiota during pregnancy.

As further evidence that the maternal microbiota can impact neurodevelopment of human offspring, epidemiological studies show a clear association between maternal infections, particularly those occurring during the first trimester of pregnancy, and ASD development in the offspring. Prominent maternal infections associated with ASD development in children include viral pathogens, such as herpes simplex virus type 2, cytomegalovirus and rubella, as well as the *Toxoplasma gondii* parasite (124–127). The impact of these microbes on the developing fetus may by driven by the vertical transfer of pro-inflammatory cytokines, induced in response to infection. Indeed, children born to mothers with chronic inflammatory diseases, such as obesity, diabetes, autoimmune diseases and asthma, also have an increased risk of neurodevelopmental disorders (128). However, it should be noted that many of these chronic inflammatory diseases and infectious pathogens are also accompanied by shifts in the composition and diversity of the gut microbiota, suggesting that the vertical transfer of microbial molecules may also impact fetal development (129, 130). Thus, dysbiosis may be one of the driving forces by which inflammatory diseases can increase the risk of neurodevelopmental disorders.

Collectively, the correlative data linking maternal immune activation and/or dysbiosis to ASD development in humans, combined with the causative role of SFB in driving the murine MIA model, suggests that dysbiosis of the maternal microbiota during gestation may contribute the risk of ASD in children.

### The Gut Microbiota Modulates Microglial Function

Microglia are highly sensitive to environmental changes, not just locally, but on a global scale. On the most basic level, microglia are readily activated in response to systemic inflammation or circulating LPS, specifically in CNS regions with fenestrated capillaries, including the choroid plexus and the circumventricular organs (131, 132). If the insult is great enough, systemic LPS challenge can trigger the activation of microglia that rapidly spreads from the circumventricular organs into the brain parenchyma, mediated by the autocrine and paracrine effects of microglial TNFα and IL-1β production (132–134). Whilst the gut microbiota will not induce systemic inflammation under homeostatic conditions, it has been well-documented that ASD is associated with barrier defects in the GI tract (135–137), often referred to as a “leaky gut”, and impaired blood-brain barrier (BBB) integrity (115, 136, 138). Thus, it is possible that inappropriate trafficking of bacterial cell wall components through the intestinal barrier to the CNS, through a permissive BBB, could contribute to abnormal microglial activation and associated neurological symptoms.

Microglia can sense peripheral changes in more subtle ways, and it is becoming increasingly apparent that they respond to distal changes in the gut microbiota composition, in the absence of overt inflammation or endotoxemia. The absence of a microbiome certainly has profound and lasting effects on microglial cell phenotype and function. Microglia development can be modulated by the maternal microbiota in a sex- and time-dependent manner (**Figure 1**). Embryonic microglial cells isolated from the offspring of GF dams exhibited marked and sex-specific differences in transcriptional profiles, increased density and ramification of embryonic microglia in different brain regions and altered chromatin accessibility compared to those from the offspring of SPF dams (139). Transcriptional differences were first apparent in the microglia of GF offspring at E14.5, with 19 differentially expressed genes differentiating GF and SPF microglia at this time. By E18.5, the transcriptional profile of microglia from male, but not female, GF embryos was profoundly distinct from their SPF counterparts, with a total of 1216 genes differentiating between male embryonic microglia from GF and SPF mice, compared to the 20 genes that differentiated between microglia from the two groups of females (139). Interestingly, most of these genes were upregulated in microglia from SPF compared to GF embryos. Not only does this work provide compelling evidence that the maternal microbiota can shape microglial cell development and maturation during pre- and perinatal stages, but the sex-specific differences highlighted in this study could account for the male bias associated with ASD development. However, it is still debated whether there is a biological mechanism accounting for the sex-specific differences associated with ASD prevalence.

The presence of an intact, complex microbiota is also required for normal microglial cell phenotype, morphology and functionality in adulthood. Microglia are more abundant in the brains of adult GF compared to SPF mice. Moreover, they are more proliferative, and exhibit altered cell morphology, characterised by longer dendrites with increased numbers of segments, branches and terminal points (140). Phenotypically, they are less mature, with increased expression of *Spi1*, CSF1R and F4/80, and are therefore less equipped to respond to immune challenges, as demonstrated by their tempered cytokine production following LPS stimulation *ex vivo* or lymphocytic choriomeningitis viral (LCMV) infection *in vivo*. Microglia from GF mice also have a reduced ability to expand in response to LCMV compared to microglia from adult SPF mice (140). Thus, microbial signals may be required for normal microglial maturation, priming them to respond to inflammatory insults later in life; see **Table 1** for a summary of some bacteria and associated metabolites shown to affect microglia.

**Table 1:** Potential links between bacterial species and microglia development and function.

****Gut microbiota Bacterial species	Metabolites****	****Potential effects on microglia	References****
*Bifidobacterium spp*	SCFAs	Homeostatic expansion of ramified microglia	(141)
*Blautia hydrogenotrophica, Clostridium* spp.	p-cresol	Induce microglial activation and expression of microglia associated CD68 protein	(142–144)
*Clostridium butyricum*	Mainly butyrate	Attenuate microglia activation and microglia-mediated neuroinflammation	(145)
*Lactobacillus* spp.	unknown	Regulate microglial dystrophy and activation during prenatal periods	(146, 147)
Bacteroides spp, Clostridium spp.	propionate	Induce microglial activation and production of inflammatory mediators (in high concentrations)	(148, 149)

Recolonizing mice with a complex microbiota or feeding them short-chain fatty acids (SCFA) can rescue the abnormal microglial maturation associated with GF mice (140). SCFA are bacterial metabolites derived from microbial fermentation. All the three main SCFAs, propionate, butyrate and acetate, are able to cross the blood brain barrier (BBB) during steady-state through monocarboxylate transporters, and are detectable in the CSF in humans (150). Altered concentrations of SCFA are observed in faecal samples from children with ASD, and ASD-associated bacteria, such as Clostridia and Bacteroidetes, are important producers of propionate and its derivates (151–154). Further supporting a role for propionate in neuropathology, the administration of high amounts of propionate, by different routes, can dramatically increase microglial cell activation, thus increasing the local production of inflammatory cytokines that induce bystander damage and the development of ASD-like behaviours in mice (148, 150). On the other hand, butyrate promotes the transcription of genes involved in neuronal inhibitory pathways, thus improving social behavior in the BTBR mouse strain, an idiopathic model of ASD (155). Considering that butyrate shows anti-inflammatory effects in microglia, and that microglia act as important modulators of neuronal inhibitory/excitatory pathways in ASD models, it seems entirely plausible that the beneficial role of butyrate is at least partially mediated through microglial cell-modulation (156–158).

**Figure 2:**
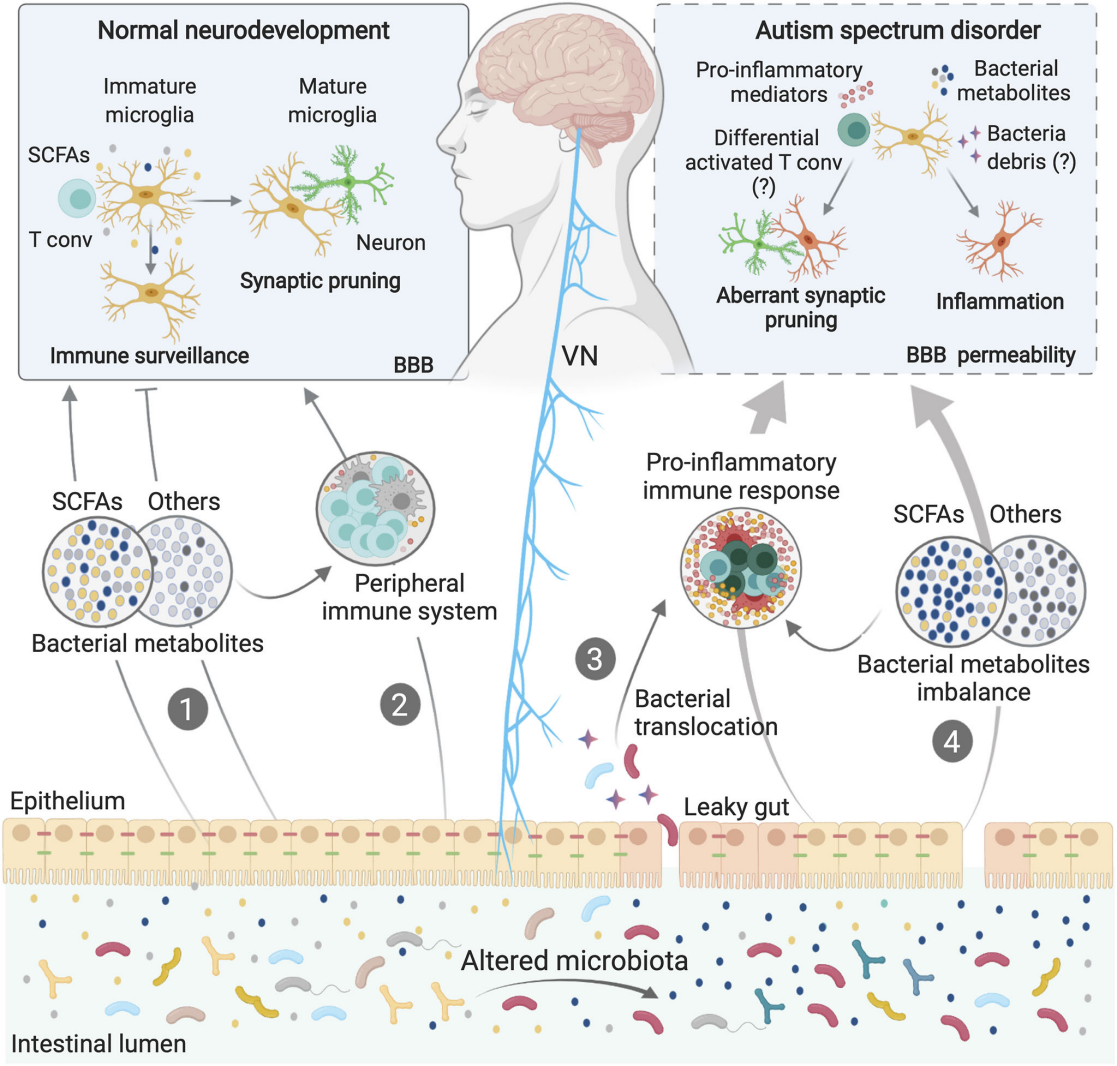
Microbiota-Microglia modulation in ASD. Microbiota-microglia communication is mediated *via* multiple direct and indirect mechanisms, including the production of bacterial metabolites, such as SCFAs (1), direct modulation of the peripheral immune system and cytokine milieu (2), and direct activation of the vagus nerve (VN) by bacterial compounds and metabolites. During homeostasis, some bacterial metabolites and components of the immune system can activate the VN or reach the brain *via* the systemic circulation, directly affecting microglial maturation and functions (1; 2). In some neurodevelopmental disorders, including ASD, dysbiosis of the gut microbiota can induce loss of gut barrier integrity. Higher intestinal permeability may allow bacterial translocation (3), as well as an imbalance in circulating bacteria-derived components (4), thus activating immune signaling pathways, including the release of cytokines and other proinflammatory molecules. Both bacterial components and proinflammatory mediators can cross the blood brain barriers (BBB) or activate the VN, inducing aberrations in the normal homeostatic functions of microglia, such as surveillance, synaptic pruning and inflammatory states, contributing to ASD symptoms.

Aberrant production of p-Cresol, a metabolite produced mainly by intestinal microbes, has been described in the fecal samples from children with ASD (142, 159). Interestingly, p-Cresol has recently been suggested induce the elevation of microglia-associated CD68 protein in the prefrontal cortex of mice with p-Cresol sulfate (PCS)-induced neuroinflammation (144). Although the specific mechanisms remain to be fully established, these data suggest that imbalances in the production of microbial metabolites might contribute to ASD pathogenesis *via* their effects on microglial cells (**Figure 2**). Notably, bacterial metabolites can be transferred from mother to fetus during gestation (160) and could thus account for the neurodevelopmental changes associated with maternal dysbiosis.

In addition to the direct effects that bacterial metabolites may have on the CNS, the immune system is a potential mediator of the gut-to-brain communication associated with ASD. Development, maturation and activation of the peripheral immune system is heavily influenced by the gut microbiota, particularly during early life (129, 161). Perturbation of the normal microbiota during this critical window, or even during pregnancy, can cause long-lasting immune alterations, conferring susceptibility to several disorders, including neurodevelopmental disorders (130, 162). Indeed, in addition to gut dysbiosis, children with ASD often present with abnormal activation of peripheral blood mononuclear cells and increased levels of systemic inflammatory mediators, including IL-1β, IL-6, CCL2, IFN-γ and IL-17 (163–166). Similarly, genetic and environmental ASD models show permanent systemic immune dysregulation and suggest a detrimental role of inflammation in the aetiology and/or maintenance of ASD (167, 168). For example, the behavioural deficits associated with the MIA model can be restored by a bone-marrow transplant from the offspring of PBS-injected control dams, thus highlighting the detrimental role the immune system can play in mediating the ASD-like symptoms associated with this model (167). It has been widely published that peripheral immune system activation can have a profound impact on brain function and behaviour (131, 169–171). Given that microglia can sense and respond to changes in circulating inflammatory mediators, it is possible that aberrant immune activation could contribute to the neuropsychiatric symptoms associated with ASD *via* effects on microglial cells (**Figure 2**).

Recent work has elucidated a novel pathway of immune-mediated microglial cell maturation whereby activated CD4^+^ T helper cells migrate to the CNS, facilitating microglial fetal-to-adult transition (172). Crucially, peripheral activation of conventional CD4^+^ T cells by the microbiome is essential to license their migration to the brain in steady state. An absence of CD4^+^ T cells in the brain, as observed in MHC class II-deficient mice, induces altered neuronal synapses and abnormal behaviour similar to those observed in some ASD models. In these mice, microglial differentiation was arrested between fetal- and adult-states. Although this study failed to address what direct effects, if any, microbial diversity might play on the phenotypes observed, it provided proof-of-principle that gut dysbiosis could impact microglial maturation in ASD patients *via* altered CD4^+^ T cell peripheral activation (172).

Finally, both gut bacteria and their metabolites, as well cytokines and other immune mediators, can directly stimulate the vagus nerve (VN), which in turn, relays information to the CNS (173–177). The VN is one of the most prominent aspects of the parasympathetic nervous system and has been extensively studied for its involvement in digestion, satiety, stress response, and regulation of inflammation (178). It also constitutes one of the main pathways of neuroimmune communication, driving sickness behaviour in response to systemic LPS challenge (171). Vagal afferent fibers innervate all the layers of the intestinal wall (178). Although it does not extend into the lumen of the GI tract, the VN is exposed to bacterial components that diffuse across the GI barrier, such as neurotransmitters, metabolites and major components of bacterial cell walls. VN neurons express numerous pattern recognition receptors, as well as receptors for SCFA and serotonin, allowing them to interact with these molecules directly (177, 179–181). The gut microbiota may also interact with the VN indirectly, by altering the inflammatory milieu of the intestine (**Figure 2**). Afferent VN fibers also express numerous cytokine and chemokine receptors (182). As such, intestinal inflammation induced by dysbiosis can be sensed by the VN and transmitted to the brain; an effect known to influence microglial activation and neuroinflammation (183, 184). Thus, by stimulating the VN, either directly or indirectly, the gut microbiota may regulate behavior in patients with ASD (**Figure 2**). Indeed, experiments in mouse models have shown that by stimulating the VN, gut microbes, such as *L. reuteri*, can improve ASD symptoms (110). Moreover, *Lactobacillus* strains can regulate behavioral and physiological responses in a manner that requires VN stimulation (173).

Collectively, these data demonstrate some of the complex pathways by which the gut microbiota can remotely modulate microglial cell function and associated behavioural changes. They also provide proof of principle that microbiome-based therapies could alleviate ASD symptoms *via* their putative effect on microglia.

### Dysbiosis, Immune Dysfunction and a Leaky Gut in ASD

Although it is yet to be fully established, a causal relationship between immune dysfunction, dysbiosis and the barrier defects associated with ASD patients seems likely, and we propose this as a major factor in the maintenance of neurological dysfunction in ASD (**Figure 2**). Dysbiosis of the intestinal microbiota could certainly induce both gut permeability and abnormal intestinal inflammation through interactions with local immune and mesenchymal cells (185). These interactions classically induce the production of a wide range of pro-inflammatory mediators, amplifying local inflammatory responses and possibly driving the GI-related co-morbidities that many ASD patients endure. As such, the intestine is a likely source of the chronic low-grade inflammation observed systemically in ASD patients (32, 186). Supporting these hypotheses, the dysbiosis observed in murine offspring exposed to the MIA model is accompanied by elevated levels of colonic IL-6 and a widespread defect in intestinal barrier integrity, all of which are restored following reconstitution with *B. fragilis* (115).

Microglia express numerous cytokine receptors, as well as toll-like receptors (TLR)-2, -4 and -7 (187–189), as does the BBB endothelium and the VN. Moreover, BBB permeability is known to increase in response to circulating cytokines. Thus, in addition to locally activating the VN, the putative release of inflammatory molecules and bacterial cell-wall components from the gut into the circulation of ASD patients could increase BBB permeability, resulting in widespread microglial cell activation and dysfunction (11, 190). In addition to the immune pathways described, dysbiosis and a leaky gut could create imbalances in circulating bacterial metabolites, which can cross the BBB to interact with microglia directly (**Figure 2**).

In summary, although a clear, causative role for the microbiota–microglia axis in ASD onset or development has yet to be fully described, the findings highlighted in this review suggest that progression of ASD may have microbial origins and thus paves the way for further research into whether therapeutic microbial manipulation could help stem the tide of increasing ASD incidence. We believe further study of this to be of fundamental importance for establishing novel prophylactic strategies that could prevent ASD.

## Conclusions

We are still unravelling the complex tri-directional relationships linking the microbiota with microglial function and ASD development. Here we describe recent evidence implicating microglia in ASD development, and discuss how environmental risk factors, particularly gut dysbiosis, could compromise the immunological and neurological functions of microglia to drive permanent changes in the brain. We have also highlighted how perturbations in the gut microbiota during prenatal and neonatal periods, induced by antibiotics, dietary changes or infections, could compromise microglial function, thus altering brain function and increasing the risk of ASD. Recent studies have begun to clarify the significant influence the gut microbiota has on microglial phenotype during steady-state, and in numerous models of neurological disorders. However, more research is required to identify the precise mechanisms by which microglia and the gut microbiota collude to drive neurodevelopmental disorders, particularly in humans. We hope that future studies, using metabolomics assays and advanced next-generation sequencing platforms, will reveal specific microbial communities or molecules associated with ASD pathogenesis or alleviating symptoms, as well as the precise molecular mechanisms involved. This could pave the way for the identification of novel treatment targets and/or the rational design of probiotics to treat or prevent ASD.

## Author Contributions

All authors listed have made a substantial, direct, and intellectual contribution to the work, and approved it for publication.

## Funding

MD-F is supported by a Beverley Phillips Postdoctoral Fellowship and a Cumming School of Medicine Postdoctoral Fellowship.

## Conflict of Interest

The authors declare that the research was conducted in the absence of any commercial or financial relationships that could be construed as a potential conflict of interest.
